# Development of U-Net Breast Density Segmentation Method for Fat-Sat MR Images Using Transfer Learning Based on Non-Fat-Sat Model

**DOI:** 10.1007/s10278-021-00472-z

**Published:** 2021-07-09

**Authors:** Yang Zhang, Siwa Chan, Jeon-Hor Chen, Kai-Ting Chang, Chin-Yao Lin, Huay-Ben Pan, Wei-Ching Lin, Tiffany Kwong, Ritesh Parajuli, Rita S. Mehta, Sou-Hsin Chien, Min-Ying Su

**Affiliations:** 1 Department of Radiological Sciences, University of California, Irvine, CA USA; 2 Department of Medical Imaging, Taichung Tzu-Chi Hospital, Taichung, Taiwan; 3 School of Medicine, Tzu-Chi University, Hualien, Taiwan; 4 Department of Radiology, E-Da Hospital and I-Shou University, No. 1, Yida Road, Jiaosu Village, Yanchao District, 8244 Kaohsiung, Taiwan; 5 Department of General Surgery, Taichung Tzu-Chi Hospital, Taichung, Taiwan; 6 Department of Radiology, Kaohsiung Veterans General Hospital, Kaohsiung, Taiwan; 7 Department of Radiology, China Medical University Hospital, Taichung, Taiwan; 8 Pasadena City College, Pasadena, CA USA; 9 Department of Medicine, University of California, Irvine, CA USA; 10 Division of Plastic Surgery, Taichung Tzu-Chi Hospital, Taichung, Taiwan; 11 John Tu and Thomas Yuen Center for Functional Onco-Imaging, University of California, 164 Irvine Hall, Irvine, CA 92697-5020 USA

**Keywords:** Breast segmentation, Deep learning, Fibroglandular tissue segmentation, Transfer learning, U-net

## Abstract

To develop a U-net deep learning method for breast tissue segmentation on fat-sat T1-weighted (T1W) MRI using transfer learning (TL) from a model developed for non-fat-sat images. The training dataset (*N* = 126) was imaged on a 1.5 T MR scanner, and the independent testing dataset (*N* = 40) was imaged on a 3 T scanner, both using fat-sat T1W pulse sequence. Pre-contrast images acquired in the dynamic-contrast-enhanced (DCE) MRI sequence were used for analysis. All patients had unilateral cancer, and the segmentation was performed using the contralateral normal breast. The ground truth of breast and fibroglandular tissue (FGT) segmentation was generated using a template-based segmentation method with a clustering algorithm. The deep learning segmentation was performed using U-net models trained with and without TL, by using initial values of trainable parameters taken from the previous model for non-fat-sat images. The ground truth of each case was used to evaluate the segmentation performance of the U-net models by calculating the dice similarity coefficient (DSC) and the overall accuracy based on all pixels. Pearson’s correlation was used to evaluate the correlation of breast volume and FGT volume between the U-net prediction output and the ground truth. In the training dataset, the evaluation was performed using tenfold cross-validation, and the mean DSC with and without TL was 0.97 vs. 0.95 for breast and 0.86 vs. 0.80 for FGT. When the final model developed with and without TL from the training dataset was applied to the testing dataset, the mean DSC was 0.89 vs. 0.83 for breast and 0.81 vs. 0.81 for FGT, respectively. Application of TL not only improved the DSC, but also decreased the required training case number. Lastly, there was a high correlation (*R*^2^ > 0.90) for both the training and testing datasets between the U-net prediction output and ground truth for breast volume and FGT volume. U-net can be applied to perform breast tissue segmentation on fat-sat images, and TL is an efficient strategy to develop a specific model for each different dataset.

**Supplementary Information:**

The online version contains supplementary material available at 10.1007/s10278-021-00472-z.

## Introduction

Breast MRI is a well-established clinical imaging modality for management of breast cancer. In addition to its use in diagnosis and pre-operative staging, breast MRI is also recommended for annual screening in women with a high risk of developing breast cancer [1], as a complementary exam to mammography which has limitations in cases of high breast density. Furthermore, many states in the USA have passed the breast density notification law which has raised awareness and led to the increased clinical use of breast MRI [2, 3]. As a result, this has led to the fast accumulation of a large breast MRI database, which can be used for exploring the clinical use of quantitative breast density. There are two potential clinical applications, one for improving the accuracy of risk-prediction models [4, 5], and the other for evaluating the response to different treatments, such as hormonal therapy [6] and neoadjuvant chemotherapy [7].

Many semi-automatic and automatic computer-aided methods have been developed for segmentation of breast and fibroglandular tissue (FGT) [8–13]. However, operator interventions and post-processing manual corrections may be needed, which are subjective and time-consuming. Therefore, despite great progress, efficiency and accuracy need to be further improved for standard clinical use of MR-measured density. A fully automatic method that can achieve a high accuracy will be extremely helpful for exploring and implementing the application of quantitative breast density in clinical settings.

Machine learning using convolutional neural networks (CNNs) provides an efficient method in imaging processing, which has been applied in image classification, object recognition, and medical image analysis for various clinical tasks [14–16]. Dalmış et al. first applied deep learning for breast MRI segmentation and demonstrated improved efficiency over an atlas-based method [11]. In a recent study, we reported an automatic segmentation method using the Fully-Convolutional Residual Neural Network (FC-RNN), commonly noted as U-net, for tissue segmentation on non-fat-sat T1-weighted (T1W) MRI [8], which could achieve a high accuracy.

For diagnosis of breast cancer using MRI, the fat-sat images were utilized more often than non-fat-sat images. In a recent survey distributed among 189 members from more than 20 countries by the European Society of Breast Imaging (EUSOBI) board, it was noted that not only were fat-sat sequences preferred over non-fat-sat sequences, but 77% preferred using only the fat-sat T1W sequences alone [17]. Fat suppression, however, is a challenging issue in breast MR imaging as it will also affect the breast density measurements [18]. Magnetic susceptibility differences between breast tissue and air causes local magnetic field (B0) inhomogeneity, which often leads to incomplete fat suppression and artifacts [19, 20]. Fat suppression is also more difficult using 3 T versus 1.5 T MRI [20] and in breasts with a high percentage of fat or with breast implants [19]. Furthermore, the signal-to-noise ratio (SNR) is lower on fat-sat than non-fat-sat images, which makes tissue segmentation even more challenging.

The purpose of this study was to apply FC-RNN, or U-net, for segmentation of breast and FGT on fat-sat images. Two datasets from different hospitals were used, one for training and the other for independent testing. In addition, the benefit of transfer learning (TL) was investigated. Our previous model developed for segmentation of non-fat-sat images was used as the basis and re-trained for fat-sat images. The results obtained without and with TL were compared.

## Materials and Methods

### Subjects

Three datasets were used in this retrospective study. The non-fat-sat dataset from 286 patients was from a previous study [8]. Our fat-sat training dataset had 126 women (mean age 48.5 years old, range 22–67 years old) with unilateral cancer, while the fat-sat testing dataset had 40 women (mean age 44 years old, range 33–70 years old) from another medical institution, also with unilateral cancer. Magnetic resonance imaging was performed for diagnosis or pre-operative staging. In this study, only the contralateral normal breast was used for segmentation.

### MR Protocols

The non-fat-sat dataset was acquired using a 3 T scanner (Trio-Tim, Siemens Medical Solutions, Erlangen, Germany), with only the pre-contrast T1W images without fat suppression being utilized. For the fat-sat training set, MRI was performed using a 1.5 T scanner (Magneton Skyra, Siemens Medical Solutions, Erlangen, Germany) with a 16-channel Sentinelle breast coil. Dynamic contrast-enhanced (DCE)-MRI was acquired using a fat-suppressed three-dimensional fast low angle shot (3D-FLASH) sequence with one pre-contrast and four post-contrast frames, with TR/TE = 4.50/1.82 ms, flip angle = 12°, matrix size = 512 × 512, field of view = 32 cm, and slice thickness = 1.5 mm. The spatial resolution was 0.6 × 0.6 × 1.5 mm. The pre-contrast, fat-suppressed T1W imaging sequence was used for analysis. For the fat-sat testing set, MRI was done using a 3 T scanner (Magnetom Skyra, Siemens Medical Solutions, Erlangen, Germany) with a 16-channel Sentinelle breast coil. The pre-contrast, fat-suppressed T1W imaging sequence used for density analysis was also acquired using the 3D-FLASH sequence, with TR/TE = 4.36/1.58 ms, flip angle = 10°, matrix size = 384 × 288, field of view = 30 cm, and slice thickness = 1.0 mm.

### Ground Truth Segmentation

A chest template–based algorithm was used to segment the breast area as the ground truth [9]. Within the segmented breast, the next step was to differentiate FGT from the adipose tissue. The nonparametric nonuniformity normalization (N3) combined with Fuzzy C-means (FCM) algorithms were used to correct the field inhomogeneity (bias-field) within the imaging region [21]. Then, K-means clustering was applied to segment FGT and adipose tissues on pixel levels. The segmentation results were inspected by a radiologist (JHC), with 15 years of experience interpreting breast MR images. If necessary, manual correction was done. The results were used as the ground truth for neural network training and for evaluating the segmentation accuracy.

### U-net Architecture

The goal was to use U-net to separate three-class labels on each MR image, including fatty tissue and FGT inside the breast, and all non-breast tissues outside the breast [22]. The first U-net was used to segment the breast from the entire image. Then, within the obtained breast mask, the second U-net was applied to differentiate the breast fat and FGT. Left and right breasts were separated using the centerline of the image, and a square matrix containing the normal breast was cropped and used as the input. The pixel intensity on the cropped image was normalized to z-score maps (mean = 0, and standard deviation = 1). The analysis was done using each slice as independent input.

**Fig. 1:**
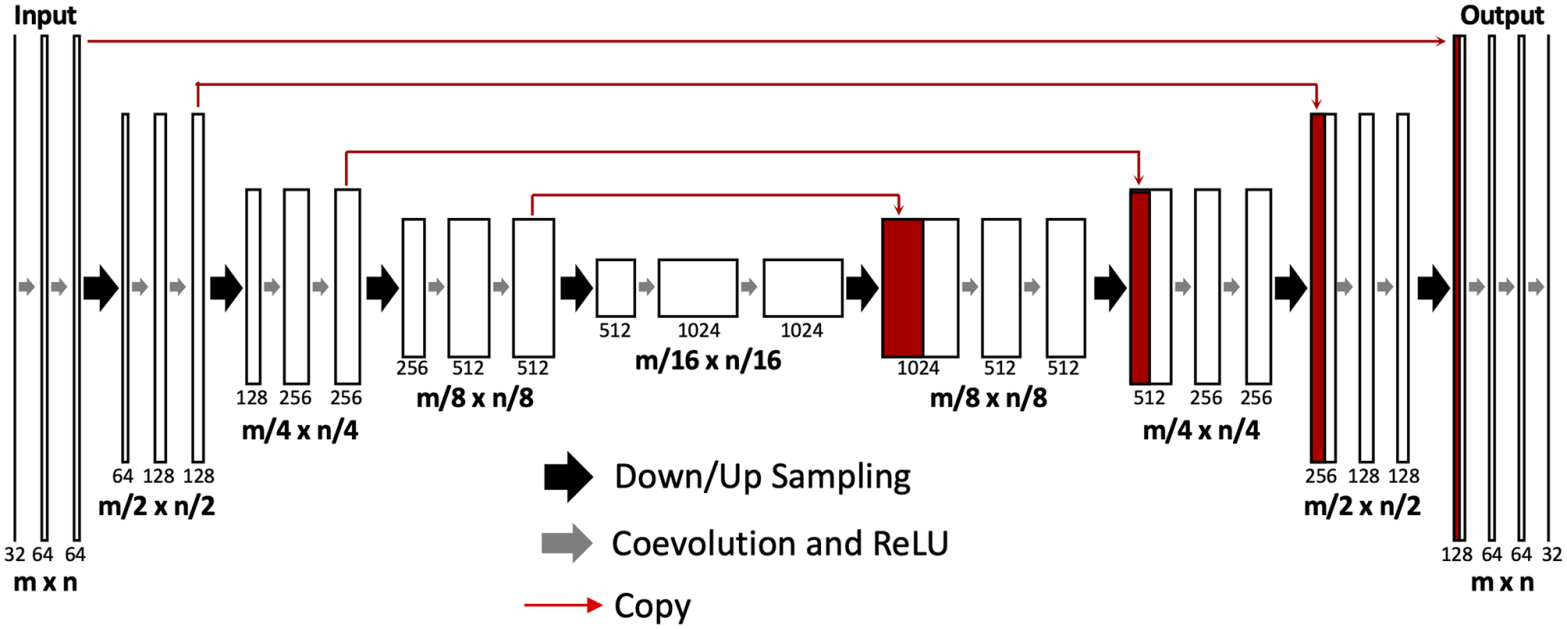
Architecture of the fully convolutional residual neural network (FC-RNN), or U-net. The U-net consists of convolution and max-pooling layers at the descending phase (the initial part of the U), the down-sampling stage. At the ascending part of the network, up-sampling operations are performed, which are also implemented by convolutions, where kernel weights are learned during training. The arrows between the two parts show the incorporation of the information available at the down-sampling steps into the up-sampling operations. The input of the network is the normalized image, and the output is the probability map of the segmentation result

The U-net architecture is illustrated in Fig. 1, with detailed methods in [8]. U-net is a popular type of FC-RNN, which is made up of convolutional and max-pooling layers at the descending part (down-sampling stage) and convolutional and up-sampling layers at the ascending part (up-sampling stage). In the down-sampling stage, the input feature map size is divided by the stride at each max-pooling layer. In the up-sampling stage, the input feature map size is increased by the up-sampling operations, which are performed and implemented by convolutions.

In this study, there were four down-sampling and four up-sampling blocks. In the down-sampling blocks, two convolutional layers with a kernel size of 3 × 3 were each followed by a rectified-linear unit (ReLU) for nonlinearity, and then followed by a max-pooling layer with 2 × 2 kernel size [23]. In the up-sampling blocks, the image was up-convolved by a factor of 2 using nearest neighbor interpolation, followed by a convolution layer with a kernel size of 2 × 2. The output of the corresponding down-sampling layer was concatenated. Next, two convolutional layers, each followed by a ReLU, were applied to this concatenated image. During the training process, the optimizer was Adam with a 0.001 learning rate [24]. Finally, a convolutional and a sigmoid unit layer were added to yield probability maps for each class which corresponded to the input image size. A threshold of 0.5 was used to determine the final segmented FGT. A maximum of 60,000 iterations were set for training, and L2 regularization was used to prevent overfitting. Software code for this study was written in Python 3.5 using the open-source TensorFlow 1.0 library (Apache 2.0 license) [25]. Experiments were performed on a GPU-optimized workstation with a single NVIDIA GeForce GTX Titan X (12 GB, Maxwell architecture).

### Transfer Learning and Evaluation

The weights of the trained model using the 286 non-fat-sat images were saved, as the initial model to re-tune parameters for training the fat-sat images with TL [26]. For comparison, another model was trained directly using the He initialization method, which is a popular method commonly used for CNN training [27]. As the initial weights differ in range depending on the size of the layers, the He method provides a controlled initialization for faster and more efficient gradient descent. In the 126 patient training set, the segmentation performance was evaluated using tenfold cross-validation. The ground truth of each case was used to evaluate the segmentation performance by calculating the dice similarity coefficient (DSC) and the overall accuracy based on all pixels. Then, a final model was developed using the hyperparameters optimized from the tenfold cross-validation runs in the training dataset and applied to the independent testing dataset of 40 patients. To evaluate the training efficiency of the TL, models were developed using different numbers of training cases (10, 20 … 110, to 126), and the obtained results were compared. Each developed model was applied to the testing dataset to obtain corresponding DSCs. In addition, Pearson’s correlation was applied to evaluate the correlation of breast volume and FGT volume between the U-net prediction output and the ground truth.

## Results

### Effect of Transfer Learning from Non-Fat-Sat to Fat-Sat Training Images

**Fig. 2:**
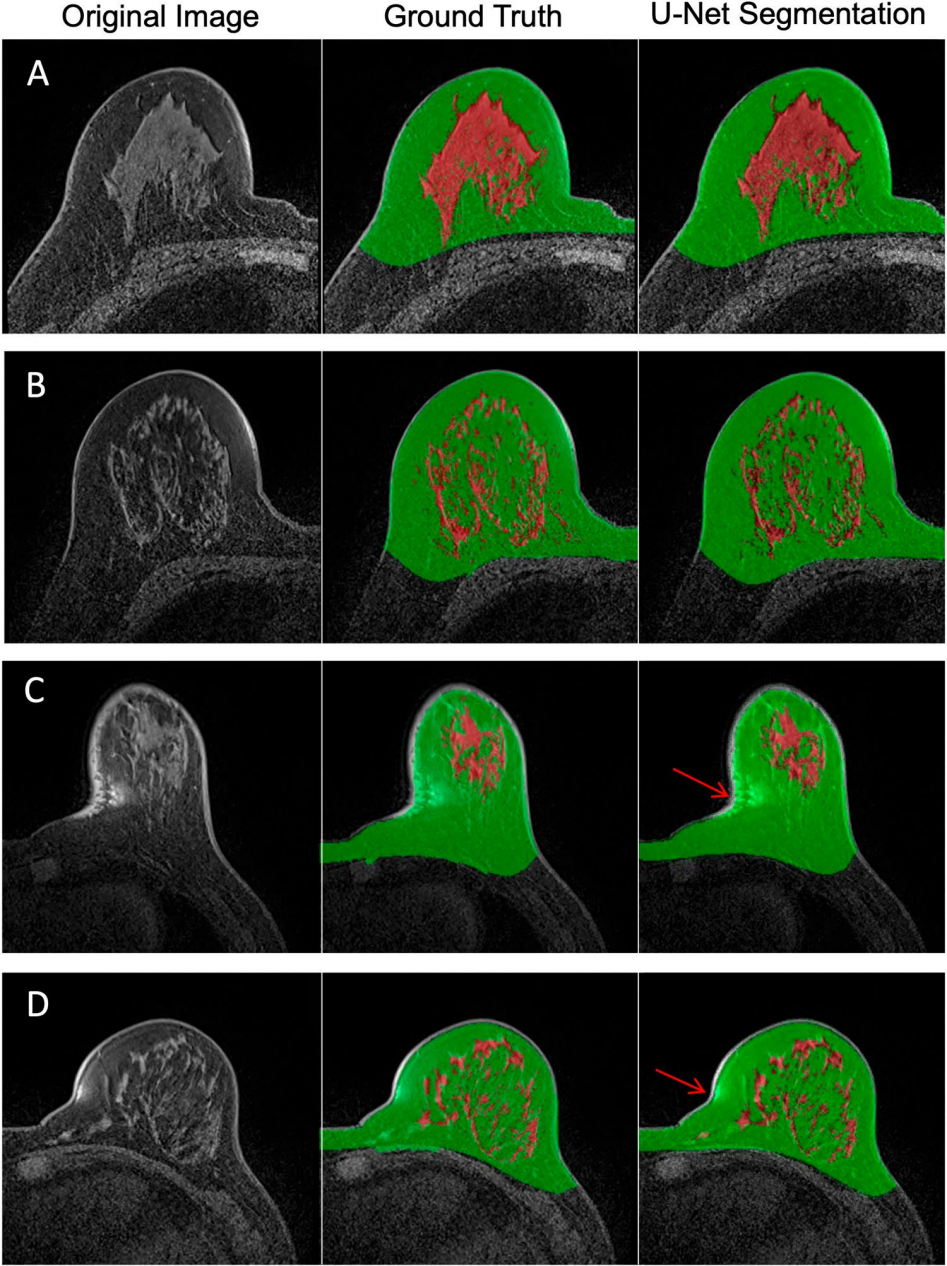
**A**–**D** Four representative cases of different breast size and parenchymal pattern showing accurate FGT segmentation using U-net compared to the ground truth. Left column: original image; central column: ground truth of breast and FGT segmentation; right column: segmentation results using U-net. Lower two panels (**C** and **D**) show two cases with susceptibility artifacts. Despite of the artifact of bright signal intensity (arrows) similar to FGT, U-net can still recognize and exclude it

Figure 2 illustrates the segmentation results from four women with different breast morphology and density. The original T1W fat-sat image, the ground truth segmentation performed using the previously developed method, and the U-net segmentation results are shown. The FGT segmentation results were very similar between the ground truth and U-net. By direct training using the He initialization without TL, the mean DSC in the tenfold cross-validation for breast segmentation was 0.95 ± 0.03. The range in the tenfold runs was 0.94–0.97, suggesting that the model was robust and could achieve a high accuracy in all runs. For pixel-based analysis, the mean accuracy was 0.97 ± 0.04 (tenfold run range 0.95–0.98). For FGT segmentation, the mean DSC was 0.80 ± 0.11 (range 0.75–0.89) with a mean accuracy of 0.86 ± 0.03 (range 0.81–0.90).

**Fig. 3:**
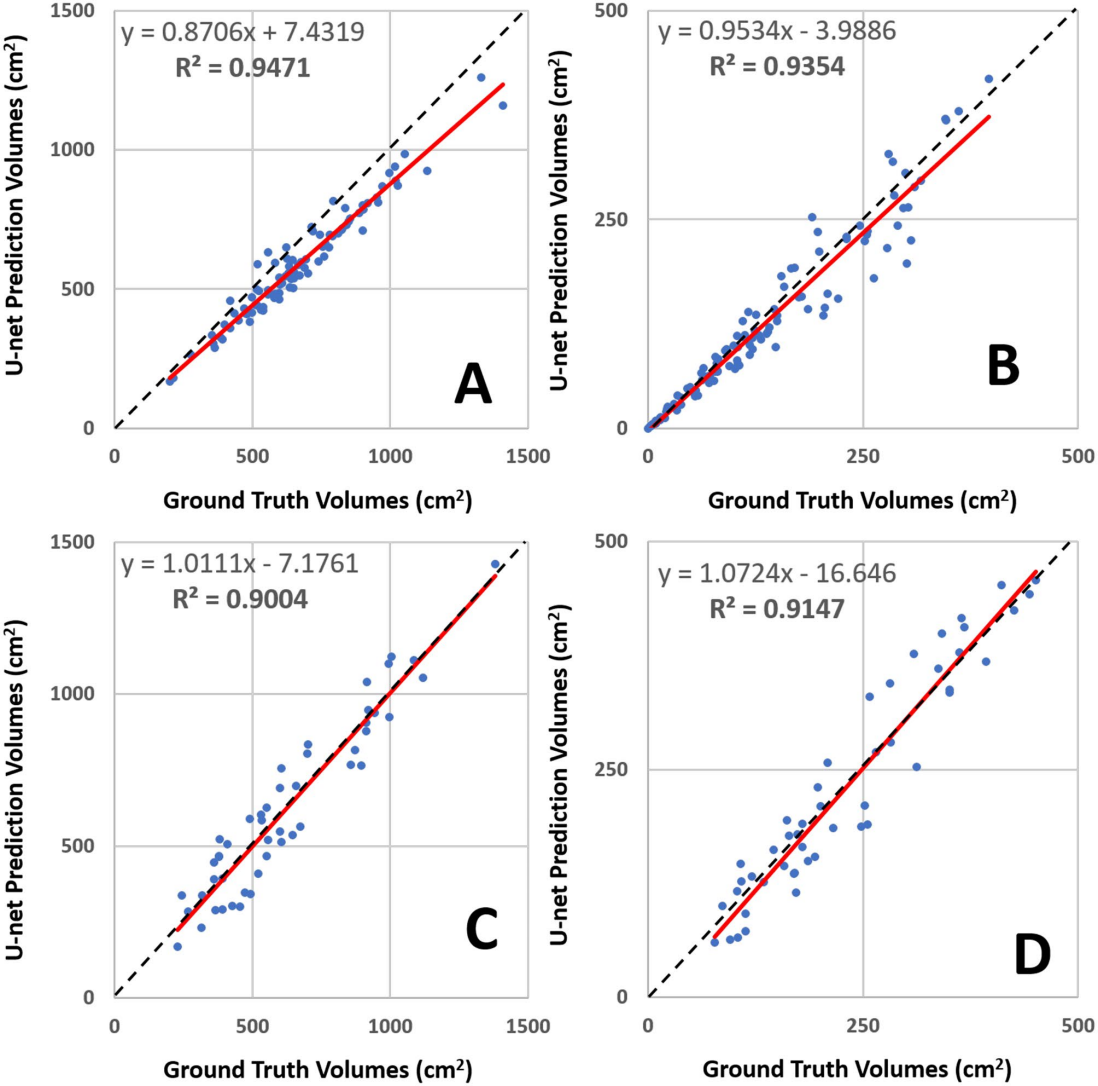
Correlation of breast volume between the ground truth obtained from the template-based segmentation method and the U-net prediction. **A** Training data breast volumes. **B** Training data FGT volumes. **C** Testing data breast volumes. **D** Testing data FGT volumes. The red line is the trend line, and the dashed black line is the unity line as reference

**Fig. 4:**
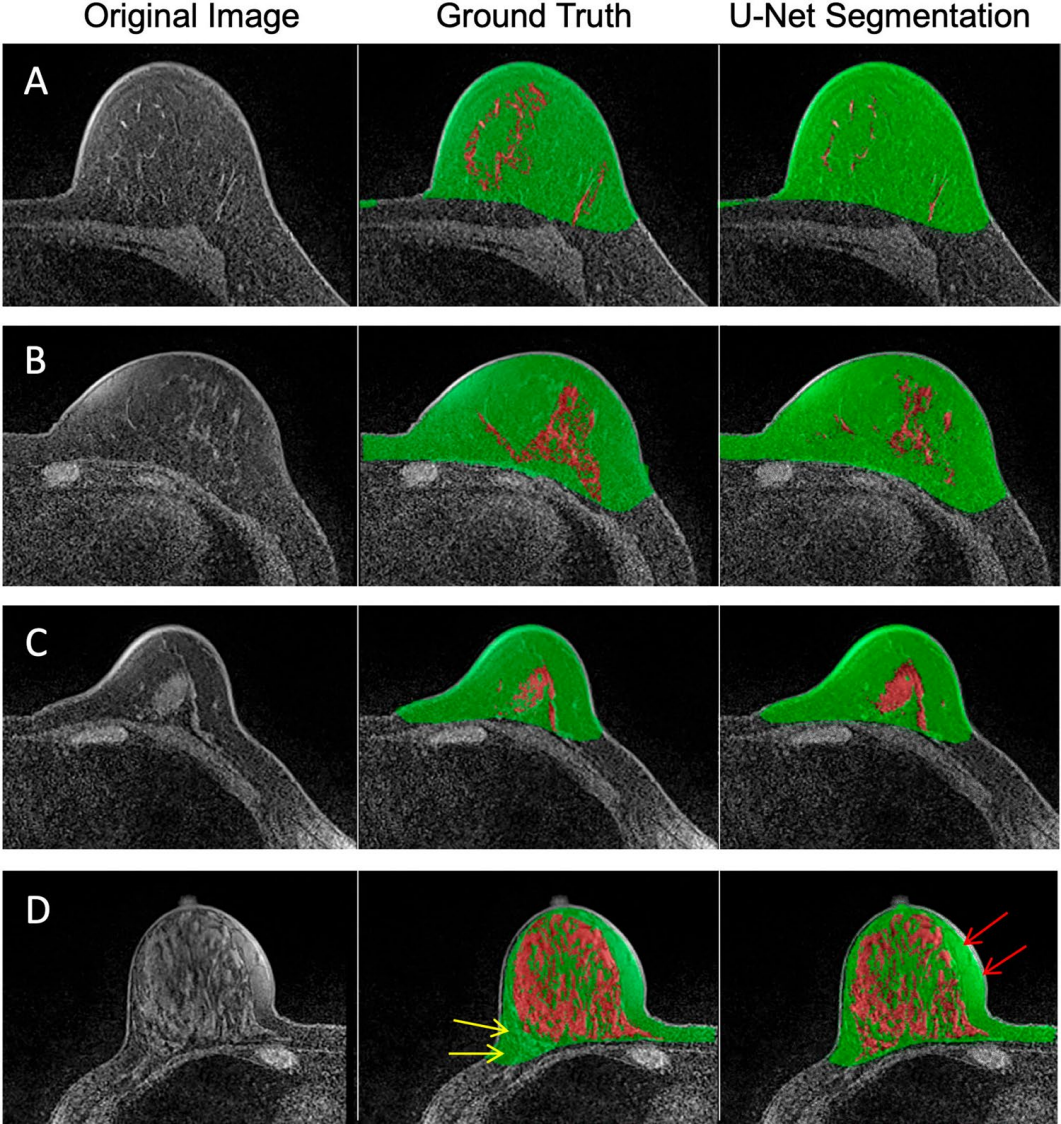
**A**–**D** Four cases of inconsistent FGT segmentation between U-net and the ground truth. Left column: original image; central column: ground truth of breast and FGT segmentation; right column: segmentation results using U-net. **A** and **B** cases show that the FGT results from ground truth are over-segmented compared to the original image. The results clearly show the superior accuracy of U-net. **C** and **D** cases show that the FGT results of the ground truth are under-segmented compared to the original image. Note the under-segmented FGT in the lower margin (yellow arrows) of the **D** case. Note also the incomplete suppression of the fat signals (red arrows) which are recognized and excluded by U-net

Model performance improved when the non-fat-sat sequence was used for initialization. For breast segmentation, the mean DSC was 0.97 ± 0.02 (range 0.96–0.98) with a mean accuracy of 0.97 ± 0.01 (range 0.96–0.97). For the FGT segmentation, the mean DSC was 0.86 ± 0.08 (range 0.74–0.90) with a mean accuracy of 0.90 ± 0.05 (range 0.87–0.96). All segmentation results are summarized in Table 1 for comparison. There is a high variation in the analyzed patients, and the range of DSC and accuracy in all patients is also included in the table. The correlation between the U-net prediction output and ground truth for breast volume and FGT volume is shown in Fig. 3. As noted, there was a high correlation (*R*^2^ > 0.90) for both the training and testing datasets. However, when carefully comparing the segmentation results case by case, we did see a mild degree of inconsistency between U-net and ground truth in some cases. Figure 4 shows four women with inconsistent segmentation results of FGT between U-net and ground truth.

**Table 1:** Segmentation dice similarity coefficient (DSC) and pixel-based accuracy in training and testing datasets without and with transfer learning

Dataset/methods	Segmentation Region	DSC		Accuracy	
		Range*	Mean ± stdev	Range*	Mean ± stdev
Training set (no transfer learning)	Breast	0.92–0.99	0.95 ± 0.03	0.93–0.99	0.97 ± 0.04
	Fibroglandular Tissue	0.44–0.92	0.80 ± 0.11	0.51–0.93	0.86 ± 0.03
Training set (w/ transfer learning)	Breast	0.96–0.99	0.97 ± 0.02	0.95–0.99	0.97 ± 0.01
	Fibroglandular Tissue	0.33–0.96	0.86 ± 0.08	0.53–0.98	0.90 ± 0.05
Testing set (no transfer learning)	Breast	0.69–0.98	0.83 ± 0.06	0.79–0.98	0.89 ± 0.03
	Fibroglandular Tissue	0.34–0.95	0.81 ± 0.10	0.52–0.98	0.87 ± 0.07
Testing set (w/ transfer learning)	Breast	0.72–0.98	0.89 ± 0.06	0.82–0.98	0.91 ± 0.03
	Fibroglandular Tissue	0.38–0.97	0.81 ± 0.08	0.48–0.98	0.86 ± 0.05

^*^Range is the value in the 126 patients in the training dataset and 40 patients in the testing dataset

### Segmentation Performance in Testing Dataset

When the developed model from the fat-sat training dataset without TL was applied to the testing dataset, the mean DSC for breast segmentation was 0.83 ± 0.06, with a mean accuracy of 0.89 ± 0.03. For the FGT segmentation, the mean DSC was 0.81 ± 0.1 with a mean accuracy of 0.87 ± 0.07. In contrast, when the model developed with TL was applied, the performance in the testing dataset was slightly improved for breast segmentation, showing mean DSC of 0.89 ± 0.06 and a mean accuracy of 0.91 ± 0.03. For the FGT segmentation, the mean DSC was 0.81 ± 0.08 with a mean accuracy of 0.86 ± 0.05.

### Efficiency of Transfer Learning

**Fig. 5:**
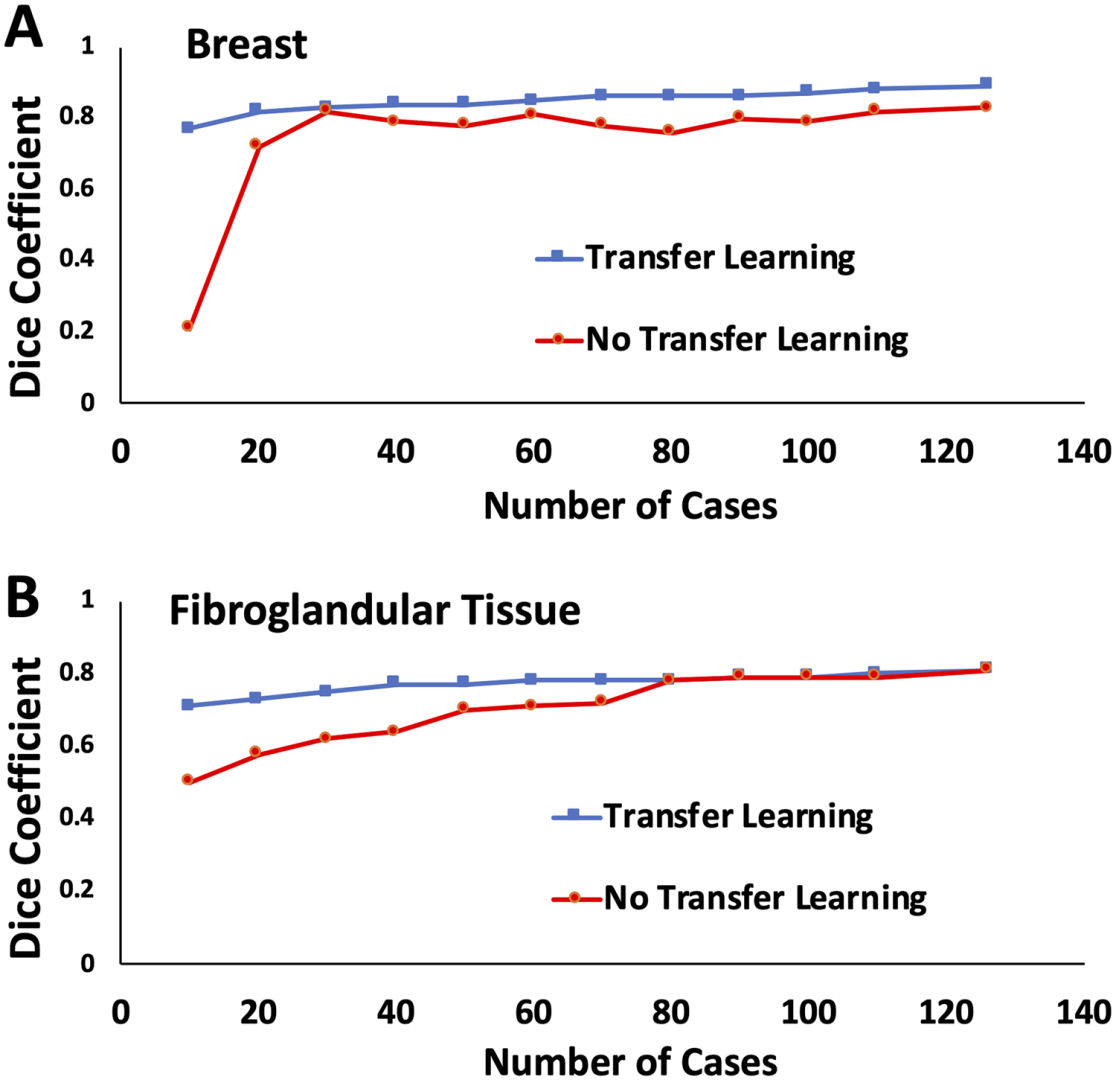
The plot of DSC in the testing dataset by using the model developed with different number of training cases from 10, 20, … to 126, with and without TL. When the training case number is small, DSC is low. When sufficient number of cases is used for training (> 30 or breast segmentation, and > 80 for FGT segmentation), the achieved DSC with and without TL is comparable, only slightly better with TL for breast segmentation

To evaluate the efficiency of training with and without TL, the performances of models developed using different numbers of training cases, 10, 20 … to 126, were compared. The results are shown in Fig. 5. Without TL, DSC was low when the training case number was small. When sufficient number of cases was used for training (> 30 or breast segmentation and > 80 for FGT segmentation), the achieved DSC could reach the level of those trained with TL. The results were only slightly lower for breast segmentation and the same for FGT segmentation.

## Discussion

In this study, we applied U-net to segment the breast and FGT on fat-sat MR images. Separate independent datasets from different hospitals were used for training and testing. A model developed previously for a non-fat-sat image dataset was used as the basis for re-training to investigate the benefit of TL [8]. Transfer learning is a popular approach in deep learning where pre-trained models are used as the starting point on computer vision tasks [26]. The results showed that the DSC for breast segmentation was very high in the training dataset, with a mean of 0.95 without TL and 0.97 with TL. In the testing dataset, the DSC was also satisfactory, with a mean of 0.83 without TL and 0.89 with TL. In contrast, FGT segmentation was more difficult compared to the breast segmentation and resulted in an overall lower DSC. In the training dataset, the mean DSC was 0.80 without TL and 0.86 with TL. In the testing dataset, the mean DSC was 0.81. The results suggested that TL could be applied to improve the segmentation accuracy compared to the direct training using the He initialization method [27]. In particular, with TL, the training efficiency could be improved without requiring a large number of input data to get a satisfactory performance. These results suggest that when the number of training cases is limited, applying TL can help to develop a good model and achieve higher accuracy.

In recent years, machine learning has been widely applied for organ/tissue segmentation on MRI, including breast and FGT segmentation. Wang et al. applied support vector machine (SVM) algorithm to T1W, T2W, proton density (PD), and Dixon sequences and obtained overlap ratios around 93–94% for FGT segmentation [10]. Although the results are good, the requirement of 4 different MR sequences is not practical in the clinical breast MRI protocol. Convolutional neural network (CNN) has become an important tool in the image processing and computer vision research. Among the different approaches, U-net is a powerful algorithm which can extract different classes of information related to different tissues in a large field, thus making it suitable for breast segmentation [22, 28]. It has been applied for breast and FGT segmentation on non-fat-sat images [11–13, 28]. Dalmış et al. [11] segmented breast and FGT using a dataset of 66 pre-contrast T1W MRI. The U-net was trained for two 2-class classification to sequentially separate the breast first, followed by fat, and then FGT, as well as one 3-class classification to segment breast, fat, and FGT simultaneously. The average DSC values for FGT segmentation obtained from the 3-class classification, two 2-class classification, and atlas-based methods were 0.850, 0.811, and 0.671, respectively, demonstrating the superior performance of U-net over the atlas-based method. This study did not have independent testing datasets. In our previous study [8], the mean DSC was 0.95 for breast and 0.91 for FGT segmentation in the training dataset, and when the model was applied to the independent testing datasets acquired with 4 different MR scanners, the mean DSC was 0.86 for breast and 0.83 for FGT segmentation.

All these studies showed consistent results, demonstrating the good performance of U-net for segmentation on non-fat-sat MR images, which had higher SNR, higher tissue contrast, and fewer image artifacts compared to fat-sat images, which made it easier for segmentation. There were few studies reporting the application of CNN for FGT segmentation on fat-sat MR images. Fashandi et al. [28] used 70 patients with fat-suppressed and non-fat-suppressed MR to train various U-net models to segment the breast but did not go further to segment the FGT within the breast. Similarly, very high DSCs were obtained for breast segmentation, with the highest of 0.96 when multi-channel inputs combing all images were used in 3D convolutions in U-net. Ha et al. [13] applied 3D U-net to segment sagittal view fat-suppressed T1W images of 137 patients and achieved a DSC of 0.95 and 0.81 for breast and FGT segmentation, respectively. The reported DSCs of breast and FDT segmentation were similar to our results even though the U-net developed by Ha et al. utilized 3D convolutions while our presented method use 2D convolutions. Meanwhile, this study focused on cross-validation of training dataset and did not have independent testing datasets.

In a study by Chang et al. [29], FGT segmentation was performed using a computer-assisted clustering method on 38 patients with both fat-sat and non-fat-sat images and showed a 5% difference in the segmented FGT volume on average. This result is not surprising, due to the different image quality and tissue contrast. The quality of fat-sat images might be affected by many factors, including MR systems (such as magnetic field strength, transmitting RF field inhomogeneity or inaccuracy, B_1_ shimming, receiver breast coil, fat-sat pulse sequence) and the variation in different patients (body shape, breast size, tissue composition, etc.). In general, any factor leading to signal variability can result in tissue misclassification, which leads to inaccurate FGT segmentation [20]. In our FGT segmentation results, although the mean DSC was greater than 0.8, the range was pretty wide, with the lowest between 0.3 and 0.4. These extreme cases had poor image quality and low SNR, which often led to low tissue contrast between fat and FGT, making it difficult to differentiate. In these cases, the clustering algorithm also had difficulty differentiating and segmenting tissues. As a result, the clustering algorithm might not provide an accurate ground truth, and the low DSC should not be interpreted as failure of the U-net. For breast segmentation, some cases also had a low DSC in the range of 0.7. For extremely fatty breast with a good fat suppression, the low SNR can make breast tissue indifferentiable from the background, as demonstrated in [28]. Despite these problems, for diagnostic purposes, fat-sat imaging is more popular than non-fat-sat imaging since enhanced tumors can be easily identified without the additional work of generating subtraction images [17]. The capability of an efficient and accurate method for segmentation of breast and FGT on fat-sat images will provide helpful information to explore its clinical application in improving the accuracy of risk prediction models [4, 5] and evaluating therapy response [6, 7].

In this study, we found several advantages of U-net for breast and FGT segmentation. First, the results of U-net were very close to ground truth in both the training and testing datasets. Second, it was found that U-net can identify several imaging problems, such as incomplete fat suppression and susceptibility artifact, and avoid the inclusion of these areas as FGT. In the past, when using different semiautomatic and automatic segmentation methods, these problems were very troublesome and in most cases required a post-processing manual correction. This made the segmentation procedure time consuming. Third, in a detailed slice-by-slice comparison of the ground truth and U-net results with the original images, we noted that in some cases, U-net outperformed the ground truth (Fig. 4). This finding raises the issue of whether basing the ground truth on operator-defined results is accurate. Therefore, artificial intelligence (AI) approaches may have tremendous potential for future application in the field of MR breast density quantification.

There were some limitations in this study. First, only two datasets, each acquired using a consistent breast MRI DCE sequence, were analyzed. The trained model may not be applicable to images acquired using a different MRI system or with a different imaging protocol. However, as demonstrated here, for future application in other datasets, the model developed in this study can be used as the basis for TL to develop a specific model for each dataset. Another limitation was the implementation of U-net based on 2D slices. To fully utilize the morphological information, 3D convolution should be employed. However, the 3D analysis will need many more trainable parameters which require more training cases.

## Conclusion

In summary, this study applied U-net to segment breast and FGT on fat-sat T1W MRI, which is a more popular imaging sequence used for diagnosis of breast cancer than the non-fat-sat sequence. The results showed that U-net could provide a fully automatic method and achieve a high DSC for breast and FGT. Furthermore, segmentation performance can be improved by applying TL which we used on a previously developed model for non-fat-sat images. The results showed that the greatest benefit of TL was to improve training efficiency and reach a satisfactory performance even with small training datasets. This can be very helpful in future clinical implementation when re-training is needed for a different dataset, e.g., acquired by a different protocol or a different MR system. The capability to measure quantitative breast volume and FGT volume on fat-sat MRI can provide a great tool for exploring the clinical application of quantitative breast density in cancer risk prediction and therapy response evaluation.

## Supplementary Information

Below is the link to the electronic supplementary material.

Supplementary file1 (DOCX 40 KB)
