# Supplementary material for: Development of U-Net Breast Density Segmentation Method for Fat-Sat MR Images Using Transfer Learning Based on Non-Fat-Sat Model
Source: J Digit Imaging. 2021 Jul 9;34(4):877–87. doi: 10.1007/s10278-021-00472-z (PMC8455741; doi:10.1007/s10278-021-00472-z)
Supplement: Supplementary file 1 — Supplementary file1 (DOCX 40 KB) [file 10278_2021_472_MOESM1_ESM.doc]

July 16, 2020

Dear Editor,

This letter is to certify that our manuscript, entitled “**Development of U-Net Breast Density Segmentation Method for Fat-Sat MR Images Using Transfer Learning Based on Non-Fat-Sat Model***”* is only submitted to ***Journal of Digital Imaging.***

I confirm that the article is not under consideration for publication elsewhere. I also confirm that each author has participated sufficiently to take public responsibility for its content.

All authors have made substantial contributions to (1) the conception and design of the study, or acquisition of data, or analysis and interpretation of data, (2) drafting the article or revising it critically for important intellectual content, (3) final approval of the version to be submitted.

The manuscript has been approved by all authors. The authors have no conflicts of interest to disclose.

With Best Regards,

Jeon-Hor Chen, M.D.
